# Ground-Based Analogs for Human Spaceflight

**DOI:** 10.3389/fphys.2020.00716

**Published:** 2020-06-23

**Authors:** Meenakshi Pandiarajan, Alan R. Hargens

**Affiliations:** Department of Orthopaedic Surgery, Altman Clinical and Translational Research Institute, University of California, San Diego, San Diego, CA, United States

**Keywords:** bed rest, head down tilt, dry immersion, wet immersion, unilateral lower limb suspension

## Abstract

This mini-review provides an updated summary of various analogs for adaptations of humans to the microgravity of space. Microgravity analogs discussed in this paper include dry immersion, wet immersion, unilateral lower-extremity limb suspension, head down tilt (HDT), and supine bed rest. All Earth-based analogs are imperfect simulations of microgravity with their own advantages and disadvantages. This paper compares these five frequently used microgravity analogs to offer insights into their usefulness for various physiological systems. New developments for each human microgravity analog are explored and advantages of one analog are evaluated against other analogs. Furthermore, the newly observed risk of Spaceflight Associated Neuro-Ocular Syndrome (SANS) is included in this mini review with a discussion of the advantages and disadvantages of each method of simulation for the relatively new risk of SANS. Overall, the best and most integrated analog for Earth-based studies of the microgravity of space flight appears to be head-down tilt bed rest.

## Introduction

This mini-review serves as an updated look upon the various analogs to microgravity. All established microgravity analogs discussed in this paper (dry immersion, wet immersion, unilateral lower-extremity limb suspension, head down tilt (HDT), and supine bed rest) are imperfect simulations of microgravity with their own merits and disadvantages. This paper serves to discuss new developments for each human microgravity analog as well as to compare these simulation methods to actual microgravity conditions of spaceflight. Furthermore, the newly observed risk of Spaceflight Associated Neuro-Ocular Syndrome (SANS) is included in this mini review with a discussion of the advantages/disadvantages of each method of simulation for space flight for the SANS risk. Due to format limitations, some aspects, such as metabolism and countermeasures, cannot be considered in this mini-review.

## Microgravity Analogs

**FIGURE 1:**
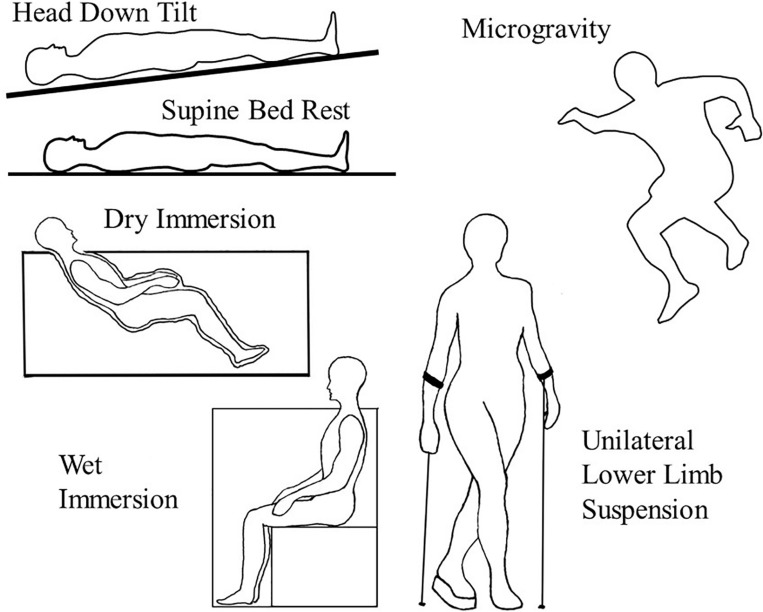
Illustrates the positions and conditions of the various microgravity analogs in Earth’s gravity as compared to actual microgravity (upper right) in which there is no weight bearing or vascular hydrostatic pressures.

Five analogs are commonly used on Earth for simulating the microgravity of space (Figure 1). The well-established microgravity analogs discussed in this paper, dry immersion, wet immersion, unilateral lower-extremity limb suspension, HDT, and supine bed rest, have their own unique advantages and disadvantages in terms of applications to various physiological systems.

## Dry Immersion

Dry immersion is a microgravity simulation analog method developed in Russia in which the subject is encapsulated in a waterproof covering and submerged, from the neck down, in water. The water is held at a standard temperature of 32–34.5°C, which is thermoneutral, and the subjects are submerged just past their clavicles (Navasiolava et al., 2010). Subjects float, simulating the effects of microgravity. This method eliminates the risks associated with extensive and prolonged exposure in water (Watenpaugh, 2016). The near complete submersion in water allows for the simulation of cardiovascular and musculoskeletal effects of microgravity (Navasiolava et al., 2010). However, as this submersion is limited to the body below the neck, it is not a perfect analog for microgravity. The fluid shift in the head and neck occurs to lesser degree than strict HDT. It also requires that subjects leave the apparatus for hygienic purposes, impacting the study, and simulated effects (Abreu et al., 2017). Because the subject is not focused upon or supported by any structure, dry immersion allows for the observation of the effects of “supportlessness.” Whereas methods such as HDT and supine bed rest redistribute stress to the posterior of the body, a lack of a support device means no loading effects are experienced the body. The effects of microgravity are demonstrated by dry immersion on a faster timeline than methods like HDT, presenting similar results to a 21-day HDT study in just 3 days (Tomilovskaya et al., 2019). 17% loss in plasma volume, comparable to loss experienced after spaceflight, is seen after 2 days of dry immersion (Treffel et al., 2017). Dry immersion also generates atrophy and loss of strength in the musculoskeletal system, with deteriorations in force and structure similar to those occurring in spaceflight during a comparable amount of time.

Dry immersion induces a head-ward fluid shift due to the hydrostatic compression of the subject. With increasing pressure with depth, lower limbs experience a higher level of head-ward fluid shift, (directing fluid to the upper body) similar to the shift seen in spaceflight. This fluid shift also decreases cardiovascular stress and heart rate decreases within hours of dry immersion. Heart rate drops by 5 bpm and blood pressure decreases by 5 mmHg in the first 4 h (Navasiolava et al., 2010). However, unlike spaceflight, subjects experience an increase in central venous pressure though there are similar changes in heart size and stroke volume. Dry immersion also shows rapid muscle and bone loss, mainly attributed to the lack of gravitational stress placed on the body (Treffel et al., 2017). Within 7 days, bone density in the lower limbs drops 2% (Navasiolava et al., 2010). When upright on Earth without dry immersion, the postural muscles of the body need to support the weight of the body and counter the effects of gravity. However, when this stress is removed, through dry immersion, muscle loss occurs, especially for stabilizing muscles of the leg and spine. It is also noted that stabilizing muscles of the lower limbs have decreased strength and force production of 20% after 7 days of dry immersion (Navasiolava et al., 2010). The rate of muscular change is rapid with dry immersion, with maximum muscle stiffness achieved within 6 h of dry immersion as opposed to several days or weeks in HDT.

Because of the near-seated position of the subject, the gravitational forces upon the body vary. This is noted in the case of where torso movement is significantly more impaired than wrist movement, suggesting a difference in the reduction of gravitational forces across the body (Wang et al., 2015).

## Wet Immersion

Water immersion requires submersion in water from the neck down. However, extensive water immersion can induce subacute dermatitis in as little as 72 h, making this method unusable for long duration studies (Willis, 1973). The benefits of a water immersion study are similar to those of dry immersion but lack the longevity required for substantial human data and wide-spread implementation (Watenpaugh, 2016). Water Immersion utilizes the hydrostatic pressure of the water environment to counteract the intravascular hydrostatic pressure gradients. However, this pressure also causes an undue influence on breathing by exerting pressure on the chest, creating a negative pressure breathing effect (Norsk, 2014).

## Unilateral Lower Limb Suspension

Unilateral lower limb suspension is one of the most cost-effective methods to study the effects of microgravity and spaceflight on the human body (Hackney and Ploutz-Snyder, 2011). The method relies on the elevation of one leg, accomplished using a singular platform shoe and crutches. This unilateral suspension allows for the continued mobility of the subject as well as an inherent control limb in the study. Increased mobility is a crucial factor in determining cost effectivity and subject compliance as semi-normal mobility allows subjects to travel, work, and remain at home. This reduces costs incurred by methods that require hospital stays or constant monitoring. While reduced monitoring means that compliance is not completely verifiable, the decreased imposition on the subject’s daily life allows for more volunteers and willing participants (Tesch et al., 2016).

This method has effects within 2–3 days of implementation, showing signs of muscle atrophy and deterioration. While this method cannot demonstrate global or cardiovascular changes from loss of hydrostatic pressures within the body, it is effective in demonstrating changes in the muscular and skeletal systems. The effects are localized to the lower leg, specifically affecting the postural muscles of the lower limb (Tesch et al., 2016). The use of lower limb suspension provides a unique benefit of coming with a built-in control. As only one leg is suspended, and thus unloaded, the other weight bearing leg and muscles can be used to evaluate the effects of the suspension. The core effect of this method, muscle atrophy, occurs at a relatively constant rate across time but does not occur uniformly across muscle fibers or individuals’ muscles. These muscular changes are consistent with the changes that occur during spaceflight, happening in similar locations and rates (Adams et al., 2003). Changes in bone density are comparable to HDT, with a 0.70% loss in bone density being observed after 21 days, similar to a 0.73% loss observed in 28-day HDT (Rittweger et al., 2006).

There is little risk to the subject aside from increased risk of venous thromboembolism relative to bed rest and spaceflight, which can be mitigated with the use of compression socks (Bleeker et al., 2004). It is recommended that individuals with risk factors, such as women on contraceptives and those with an inherited risk of deep vein thrombosis, are excluded from ULLS studies (Bleeker et al., 2004). Head-ward fluid shifts and larger possible changes to the cardiovascular or musculoskeletal system are prevented in this method by localizing the unloading to one limb, thereby preventing long lasting effects and risk to the volunteer’s health (Tesch et al., 2016). However, this localization prevents ULLS from being a valid method of study for global effects of spaceflight.

## Head Down Tilt

The focus of HDT bed rest is to induce head-ward fluid shifts. In microgravity, the body undergoes head-ward fluid shifts because hydrostatic pressures disappear, and arterial pressure is equalized throughout the body. Due to the presence of gravity on Earth, blood pressures are significantly higher in the lower limbs and feet than at the head. In order to reproduce this condition on Earth, subjects are placed in the supine position on a bed that is tilted 6 degrees to place the head closer to the ground and to elevate the feet (Hargens and Vico, 2016). Six-degrees HDT is the international standard for simulating weightlessness (Smith et al., 2011). As opposed to supine bed rest, HDT increases head-ward fluid shifts toward the head but alters the gravity vector over the entire body from anterior to posterior. This method requires sustained bed rest and a prolonged hospital stay, causing this analog to be expensive. For strict HDT, it is important that subjects do not leave this posture for short periods of time or use a pillow to prop up their heads. The use of a pillow decreases ICP (supine 14 ± 2 mmHg vs. pillow, 10 ± 2 mmHg, *P* = 0.05), which may counteract visual symptoms that might occur (Lawley et al., 2017). Removing the visual impact is not conducive to studying SANS. Eating, showering, use of urinals and bed pans must be performed in HDT posture. However, unlike lower limb suspension, HDT allows for the observation of cardiovascular effects and head-ward fluid shifts for better studies of SANS and its mechanisms and responses (Hargens and Vico, 2016; Watenpaugh, 2016; Laurie et al., 2019).

Head down tilt redistributes pressure across the posterior of the body, rather than being focused in a head to feet direction. This posture does not completely remove the application of gravity and thus, does not completely simulate the effects of a microgravity environment. By inducing HDT, the cardiovascular system no longer has to work against the force of gravity, as when standing up, mimicking a lack of gravity (Watenpaugh, 2016). Head-ward fluid shifts and cardiovascular adaptations are akin to those found in spaceflight and microgravity (Hargens and Vico, 2016). Unlike microgravity, HDT does not create a loss in tissue weight but it does induce a greater amount of fluid toward the head. The skeletal system shows a decrease in bone density, when subjected to HDT without any countermeasures, especially in the lumbar spine, pelvis, and legs. Over HDT of 2–3 months, a decrease in bone density of 3.8% was seen in the hip and a 10% decrease at the heel (Hargens and Vico, 2016). As a result of HDT, these bones no longer bear weight, thus resulting in loss of bone quality density. This analog helps identify bone regions where subjects in a microgravity environment would experience the greatest loss of bone. Furthermore, loss in bone density in the lower, now non-weight bearing limbs, can be compared to that of the upper limbs, which are not weight bearing in any position, to isolate the changes due to HDT (Hargens and Vico, 2016). Proper experimental procedure for this method requires a separate control group to attempt to accurately gauge which effects can be attributed to the microgravity simulation method.

Spaceflight Associated Neuro-Ocular Syndrome is an outcome of long-term spaceflight. The exact cause of SANS and the factors that lead to it are still under extensive research (Mandsager et al., 2016; Laurie et al., 2019). One of the widely observed results of SANS is increased choroidal thickness and increased optic disk edema. Replicating these effects on Earth has yet to be done with any analog. However, similar results to SANS in space are achieved with HDT. An extended trial may be of value, as 70-day HDT was found to show greater retinal thickening (+18 μm [+5.3%] during 70-day vs. none in 14-day) and IOP increase (+1.79 mm Hg vs. +1.42 mm Hg) than 14-day HDT (Taibbi et al., 2016; Klassen et al., 2018). In addition, hypercapnia alongside HDT has shown an increase in IOP of 0.8 mmHg (95% CI, *P* = 0.05) when compared to normal HDT (Laurie et al., 2017). However, HDT also produces a higher level of retinal thickness than that experienced in spaceflight, with a mean difference of 37 μm (95% CI, 13–61 μm; *P* = 0.005) between grounded subjects and astronauts (Laurie et al., 2019).

## Supine Bed Rest

Supine bed rest creates a uniform fluid distribution throughout the body (except for anterior to posterior fluid shifts and small hydrostatic pressure gradients) by having subjects lie in the supine position (Hargens and Vico, 2016). This method also results in the compression of posterior tissues unlike that of microgravity. In supine bed rest, the contact between the patient and the bed compresses tissues while microgravity has no such compression of tissues, e.g., during sleep. There has been little expansion in the use of this early analog for calcium balance studies. This method sometimes provides a control for HDT studies.

## Comparing Methods

All current analogs to microgravity are imperfect analogs and need to be compared to evaluate their utility for a given project or space maladaptation (Table 1). Immersion generates an even distribution of gravitational forces (Norsk, 2014). While dry and wet immersions provide similar physiological reactions to HDT, responses to immersions are more rapid than HDT. Back pain with dry immersions seems more severe than that with wet immersion and HDT. It is important to recognize that immersion relies on the neutralization of internal pressures through the pressure of water and HDT provides a head-ward fluid shift due to jugular vein congestion and slightly higher venous and arterial pressures in the head and neck. Jugular vein flow is reduced in HDT in a manner similar to space flight. While unilateral lower limb suspension is a cost-effective model for unloading of leg muscles and bones, it fails to account for the lack of gravitational stress and the head-ward fluid shifts seen in space (Tesch et al., 2016).

**TABLE 1:** This table summarizes the observable effects.

	Dry immersion	Wet immersion	Unilateral lower limb suspension	Head down tilt	Supine bed rest
Mimics cardiovascular effects of space flight	***	**	*	***	**
Mimics musculoskeletal loss of space flight	**	*	***	***	***
Feasibility of maintaining position	**	*	*	***	***
Median duration	3–7 days	4 h	30 days	30 days	30 days
Maximum duration	56 days^13.14^	12 h^13.14^	42 days^13.6^	370 days^13.14^	

* is used to indicate weak comparability, ** is used to indicate moderate comparability, and *** indicates strong comparability. Feasibility of Maintaining Position includes the physical stress placed upon the subject as well as the costs and logistics involved. ^13.6^is a reference to Hackney and Ploutz-Snyder (2011), ^13.14^is a reference to Navasiolava et al. (2010).

As it stands, no studies utilizing any method other than HDT and supine bed rest to study SANS have been published. One of the possible causes of SANS may be the increased differential between intercranial pressure and intraocular pressure that occurs in a microgravity environment (Laurie et al., 2017; Zhang and Hargens, 2018; Huang et al., 2019). Moreover, dry immersion, which may increase intracranial pressure mildly and chronically, may also be a viable method to study SANS (Arbeille et al., 2017) but the head and neck are still exposed to vascular hydrostatic pressure gradients due to gravity. Dry immersion has also been observed to increase optic nerve sheath diameter, which is linked to increased ICP (Kermorgant et al., 2017). While further studies need to be done, the best and most integrated analog for Earth-based studies of the microgravity of space flight appears to be HDT bed rest.

## Author Contributions

MP and AH contributed to the conception and creation of the manuscript. MP researched and wrote the initial draft of the manuscript. AH edited and advised on the manuscript. Both authors read and approved the submitted version of the manuscript.

## Conflict of Interest

The authors declare that the research was conducted in the absence of any commercial or financial relationships that could be construed as a potential conflict of interest.
